# Excellent outcomes of 2G-TKI therapy after imatinib failure in chronic phase CML patients

**DOI:** 10.18632/oncotarget.24478

**Published:** 2018-02-12

**Authors:** Mario Tiribelli, Massimiliano Bonifacio, Gianni Binotto, Alessandra Iurlo, Francesca Cibien, Elena Maino, Anna Guella, Gianluca Festini, Claudia Minotto, Ercole De Biasi, Federico De Marchi, Luigi Scaffidi, Luca Frison, Cristina Bucelli, Marta Medeot, Elisabetta Calistri, Rosaria Sancetta, Manuela Stulle, Nicola Orofino, Mauro Krampera, Filippo Gherlinzoni, Gianpietro Semenzato, Giovanni Pizzolo, Achille Ambrosetti, Renato Fanin

**Affiliations:** ^1^ Division of Hematology and Bone Marrow Transplantation, Department of Medical Area, ASUI Udine, Udine, Italy; ^2^ Department of Medicine, Section of Hematology, University of Verona, Verona, Italy; ^3^ Department of Medicine, Hematology Section, Padua University School of Medicine, Padua, Italy; ^4^ Hematology Division, IRCCS Ca’ Granda–Maggiore Policlinico Hospital Foundation, Milan, Italy; ^5^ Hematology Unit, Ca’ Foncello Hospital, Treviso, Italy; ^6^ Hematology Unit, Dell'Angelo Hospital, Venezia-Mestre, Italy; ^7^ Hematology Unit, Santa Chiara Hospital, Trento, Italy; ^8^ Division of Clinical Hematology, AOU Ospedali Riuniti, Trieste, Italy; ^9^ Department of Medical Specialities, Oncology and Onco-Hematology Unit, Venice, Italy; ^10^ Hematology Unit, P. Cosma Hospital, Camposampiero, Padua, Italy

**Keywords:** CML, dasatinib, nilotinib, second-line, outcomes

## Abstract

Second-generation tyrosine kinase inhibitors (2G-TKIs) dasatinib and nilotinib produced historical rates of about 50% complete cytogenetic response (CCyR) and about 40% major molecular response (MMR) in chronic myeloid leukaemia (CML) patients failing imatinib. Direct comparisons between dasatinib and nilotinib are lacking, and few studies addressed the dynamics of deep molecular response (DMR) in a “real-life” setting.

We retrospectively analyzed 163 patients receiving dasatinib (*n* = 95) or nilotinib (*n* = 68) as second-line therapy after imatinib. The two cohorts were comparable for disease's characteristics, although there was a higher rate of dasatinib use in imatinib-resistant and of nilotinib in intolerant patients.

Overall, 75% patients not in CCyR and 60% patients not in MMR at 2G-TKI start attained this response. DMR was achieved by 61 patients (37.4%), with estimated rate of stable DMR at 5 years of 24%. After a median follow-up of 48 months, 60% of patients persisted on their second-line treatment. Rates and kinetics of cytogenetic and molecular responses, progression-free and overall survival were similar for dasatinib and nilotinib.

In a “real-life” setting, dasatinib and nilotinib resulted equally effective and safe after imatinib failure, determining high rates of CCyR and MMR, and a significant chance of stable DMR, a prerequisite for treatment discontinuation.

## INTRODUCTION

The introduction and worldwide diffusion of imatinib (IM) and, subsequently, second-generation tyrosine kinase inhibitors (2G-TKIs) has dramatically improved the prognosis of chronic myeloid leukaemia (CML) patients. Long-term follow-up of the IRIS study and the German CML-IV study reported estimated overall survival (OS) rates at 10 years with IM-based therapy around 82–83% [1, 2], close to that of the general population [3]. This excellent outcome is obtained despite that, in those two studies, 40 to 50% of patients interrupted IM therapy for unsatisfactory therapeutic efficacy or adverse events (AEs), outlining the efficacy of second-line treatment.

The first 2G-TKIs introduced in the clinical practice were dasatinib (DAS) and nilotinib (NIL), which had been initially tested in CML patients failing IM. When used in chronic phase (CP), both drugs result in around 50% of sustained complete cytogenetic response (CCyR) and 40% of major molecular responses (MMR) [4, 5]. The two molecules have a favorable safety profile [6] and specific spectrum of activity against BCR-ABL1 kinase domain mutants [7].

However, due to the lack of direct comparative studies, it's unclear whether any significant differences exist in terms of short and long-term activity among the two 2G-TKIs. Aim of our study was to describe efficacy of DAS and NIL in CP-CML patients after IM resistance or intolerance in a real-life setting.

## RESULTS

### Patient characteristics

The present study included 163 CP-CML patients resistant or intolerant to IM that received either DAS (*n* = 95) or NIL (*n* = 68) as second-line therapy. Considering CML characteristic at diagnosis, the DAS and NIL cohorts were comparable for age, sex, BCR-ABL transcript type and risk scores (Sokal and EUTOS). Median duration of IM therapy was similar (DAS 19 months, range: 1–113; NIL 14 months, range: 1–149), but 27/95 patients (28%) received IM at doses >400 mg/day before DAS compared to only 9/68 (13%) before NIL (*p* = 0.03). There was a higher rate of switch to DAS than to NIL for secondary cytogenetic and/or molecular resistance (26/95, 27% vs 7/68, 10%; *p* = 0.01) while more patients changed from IM to NIL due to intolerance (31/68, 46%, vs 21/95, 22% for DAS; *p* = 0.002). Rates of primary cytogenetic and/or molecular resistance did not differ (47/95, 49% for DAS vs 28/68, 41% for NIL; *p* = 0.37), as other causes of switch (1/95, 1% for DAS vs 2/68, 3% for NIL; *p* = 0.77). Hammersmith score was almost identical in the two groups. One patient in each cohort displayed a 2G-TKI-sensitive ABL mutation at the time of IM failure, namely one M351T in a patient treated with DAS and one L364P in a patient treated with NIL (Table 1).

**Table 1:** Patients’ characteristics at diagnosis and at start of 2G-TKI

	DAS (*n* = 95)	NIL (*n* = 68)	*P*
**Age median, years (range)**	58 (18–88)	54 (20–80)	0.43
**Sex, M/F ratio**	56/39	44/24	0.56
**BCR-ABL: b_2_a_2_**	38 (40%)	30 (44%)	0.72
**b_3_a_2_**	35 (37%)	19 (28%)	0.31
**both**	16 (17%)	7 (10%)	0.23
**other/unknown**	6 (6%)	12 (18%)	0.04
**Sokal: Low**	32 (34%)	31 (46%)	0.17
**Intermediate**	42 (44%)	27 (40%)	0.68
**High**	19 (20%)	9 (13%)	0.36
**Unknown**	2 (2%)	1 (1%)	1.00
**EUTOS: Low**	81 (85%)	59 (87%)	0.97
**High**	8 (9%)	4 (6%)	0.76
**Unknown**	6 (6%)	5 (7%)	1.00
**IM therapy median, months (range)**	19 (1-113)	14 (1-149)	0.18
**IM dose escalation**	27 (28%)	9 (13%)	**0.03**
**Hammersmith score (low/evaluable)**	57/83^*^ (69%)	42/57^*^ (74%)	0.65
**Reason for 2G-TKI: Primary resistance**	47 (50%)	28 (41%)	0.37
**Secondary resistance**	26 (27%)	7(10%)	**0.01**
**Intolerance**	21(22%)	31 (46%)	**0.003**
**Other**	1 (%)	2 (3%)	0.78

### Cytogenetic and molecular responses after 2G-TKIs

Complete cytogenetic response was attained in 53/69 (77%) patients not in CCyR at the time of DAS start, compared to 27/37 (73%) patients not in CCyR at the time of NIL start (*p* = 0.81). Mean time to attain CCyR was similar (7.1 months for DAS and 5.3 months for NIL; *p* = 0.30).

Major molecular response was achieved in 52/86 (60%) patients not in MMR at the time of DAS start and in 30/50 (60%) patients not in MMR at the time of NIL start (*p* = 1). Again, mean time to MMR was not different in the DAS and NIL cohorts (12.4 vs. 8.5 months; *p* = 0.14).

Deep molecular response was attained in 39 patients with DAS (41% of the total DAS population and 75% of those achieving MMR) and in 22 patients with NIL (32% of the total NIL cohort and 73% of those with MMR) (*p* = 1).

**Figure 1:**
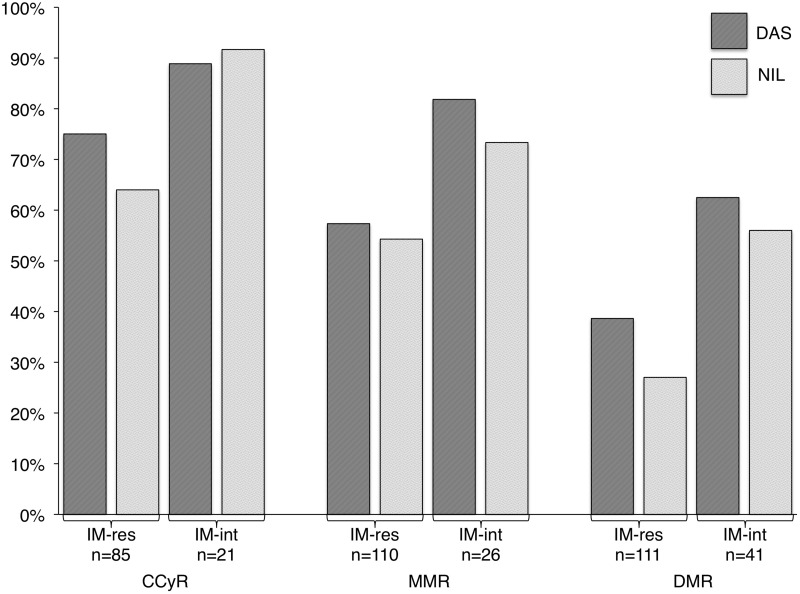
Rates of complete cytogenetic response (CCyR), major molecular response (MMR) and deep molecular response (DMR) in imatinib-resistant (IM-res) and -intolerant (IM-int) patients treated with second-line dasatinib (DAS) or nilotinib (NIL)

Patients switched to 2G-TKIs for IM intolerance had better rates of response as compared to patients switched for IM resistance: CCyR 90% vs 72% (*p* = 0.09), MMR 77% vs 56% (*p* = 0.07), DMR 59% vs 34% (*p* = 0.009) We compared cytogenetic and molecular responses obtained with DAS or NIL in IM-intolerant and IM-resistant patients separately, and we found no differences between the two 2G-TKIs (Figure 1).

### Cytogenetic and molecular responses according to 2G-TKIs dose

DAS starting dose was 140 mg once-a-day (OD) in 5 patients (5%), 100 mg OD in 79 patients (83%), and less than 100 mg OD in 11 patients (12%). NIL starting dose was 400 mg twice-a-day (BID) in 33 patients (49%), 300 mg BID in 26 patients (38%), and 200 mg BID in 9 patients (13%). Proportions of patients starting at different doses of each 2G-TKI were not different according to reason of IM failure (i.e. resistance or intolerance). Rates of cytogenetic and molecular responses were similar across different starting doses, both in DAS and in NIL cohorts (Table 2).

**Table 2:** Cytogenetic and molecular responses according to 2G-TKI starting dose

	DASATINIB (*n* = 95)			
**Starting dose**	140 mg (*n* = 5)	100 mg (*n* = 79)	<100 mg (*n* = 11)	***P***
**IM resistant/intolerant**	4/1	65/14	6/5	0.12
**CCyR**	5/5 (100%)	41/55 (75%)	7/9 (78%)	0.58
**MMR**	4/5 (80%)	43/71 (60%)	5/10 (50%)	0.62
**DMR**	3/5 (60%)	31/75 (41%)	5/11 (45%)	0.83
**Stable DMR**	2/5 (40%)	19/79 (24%)	1/11 (9%)	0.35
	**NILOTINIB (*n* = 68)**			
**Starting dose**	800 mg (*n* = 33)	600 mg (*n* = 26)	400 mg (*n* = 9)	***P***
**IM resistant/intolerant**	21/12	13/13	3/6	0.23
**CCyR**	17/21 (81%)	7/11 (64%)	3/5 (60%)	0.50
**MMR**	17/26 (65%)	8/17 (47%)	5/7 (71%)	0.44
**DMR**	12/30 (40%)	8/24 (33%)	2/7 (28%)	0.81
**Stable DMR**	6/33 (18%)	4/26 (15%)	2/9 (22%)	0.91

During follow-up, 2G-TKI dose was permanently reduced in 28/95 (29%) patients receiving DAS and 21/68 (31%) patients receiving NIL. Median time from 2G-TKI start to permanent dose reduction was 28.6 months for DAS and 10.8 months for NIL (*p* = 0.055). The main causes of permanent dose reduction were non-hematological toxicities (*n* = 30), recurrent hematological toxicities (*n* = 6), or pro-active reduction in patients with stable molecular responses and at risk for cardiovascular events (*n* = 8). Overall, 90% of patients maintained or improved over time the level of molecular response attained at the time of permanent dose reduction, without differences between DAS and NIL cohorts.

### Stable deep molecular response after 2G-TKIs

Stability of molecular response was assessable in 154 patients (DAS = 89, NIL = 65) with serial Q-RT-PCR analysis over a time-span of at least two years. The characteristics of this cohort are comparable with those of the entire population, with a median age of 55 years, a prevalence of male sex (60%), low-intermediate risk (82%), b2a2 BCR-ABL transcript (52%), a median IM duration of 19 months and resistance as main reason of switch to 2G-TKI (70%).

**Figure 2:**
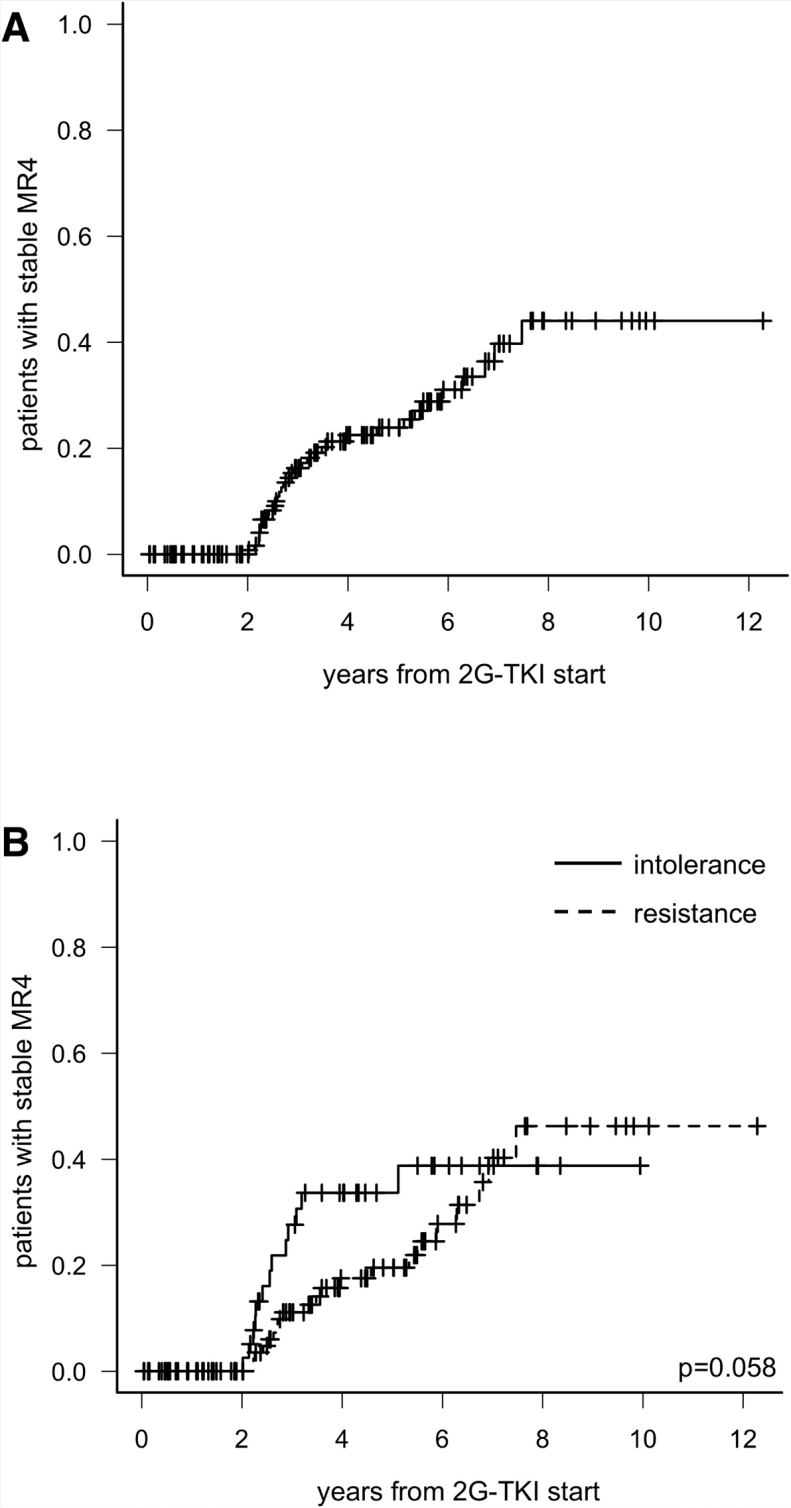
Cumulative incidence of stable deep molecular response in the whole population (**A**) and according to the reason of switch to 2G-TKI (**B**).

Eighty-three patients (53.9%) never reached a DMR, 37 patients (24.0%) achieved an unstable DMR and 34 patients (22.1%) achieved a stable DMR. The cumulative incidence of stable DMR at 5 years was 23.9% (95%CI: 15.3-31.7). (Figure 2A). Neither age, sex, risk, BCR-ABL transcript type, IM duration or 2G-TKI used correlated with the chance of attain a stable DMR; only the reason of switch to DAS or NIL was associated with such a response, as cumulative incidence of stable DMR at 5 years was 34% in intolerant patients and 19% in resistant patients (*p* = 0.058; Figure 2B). All but one patients subsequently attaining stable DMR had ≤10% BCR-ABL1 transcript level 3 months after 2G-TKIs start.

### Long-term outcomes

**Figure 3:**
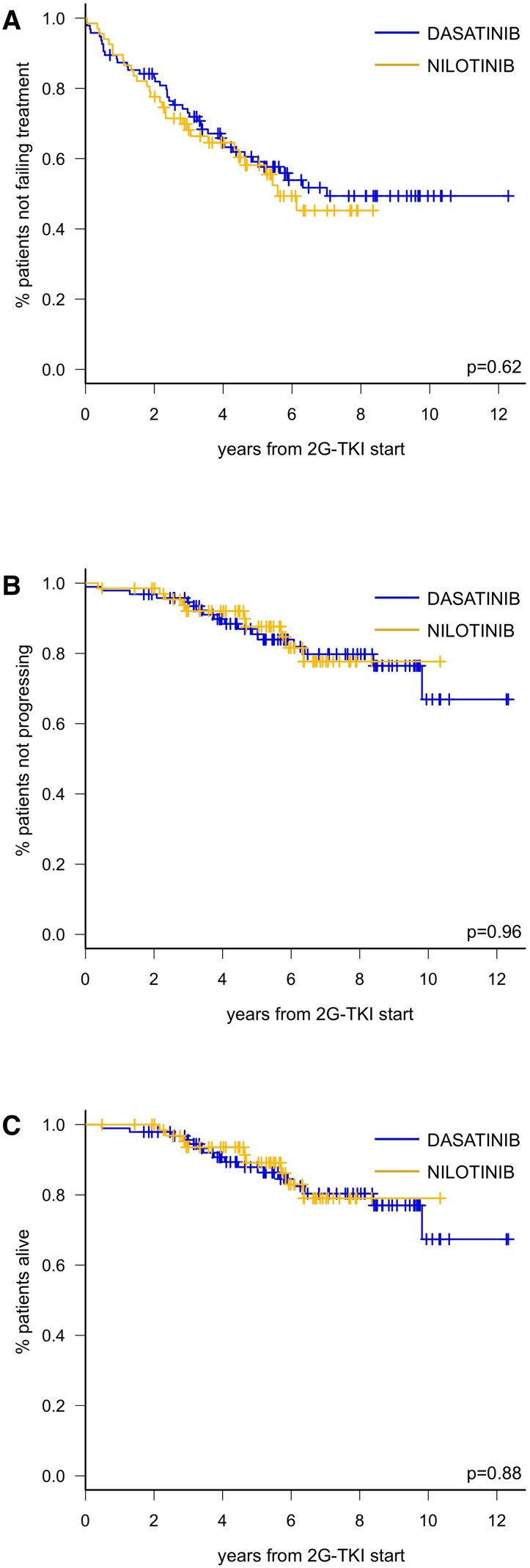
Time to treatment failure (**A**), progression-free survival (**B**) and overall survival (**C**) according to 2G-TKI treatment in CP-CML patients failing imatinib.

With a median follow-up of 48 months (range 1-147), 5-year TTF was similar for DAS (59.1%, 95%CI: 47.9-68.7) and NIL (58.1%, 95%CI: 44.5-69.5; *p* = 0.62) (Figure 3A). Forty of 95 patients (34 42%) stopped DAS due to toxicity (22/40, 55%), resistance (13/40, 32%) or other causes (5/40, 12%). The commonest AEs leading to DAS permanent discontinuation were pleural effusion (*n* = 10), heart failure (*n* = 2) and arrhythmias (*n* = 2); we recorded one case of pulmonary arterial hypertension (PAH). Thirty-two patients received third-line therapy, namely NIL (*n* = 24), IM (*n* = 2), ponatinib (*n* = 2), bosutinib (*n* = 2) or other (hydroxyurea, *n* = 2).

Twenty-six of 68 patients (38%) interrupted NIL for toxicity (14/26, 54%), resistance (9/26, 35%) or other causes (3/26, 11%). The most frequent toxicities causing permanent NIL stop were peripheral arterial obstructive disease (PAOD) (*n* = 4), cardiovascular events (*n* = 3), cutaneous adverse events (*n* = 2) and fluid retention (*n* = 2). Nineteen patients were switched to alternative therapy with DAS (*n* = 13), ponatinib (*n* = 3), or bosutinib (*n* = 3).

Five patients (4 in the NIL and 1 in the DAS group) received allogeneic stem cell transplantation for intolerance/resistance after their third-line TKI treatment (*n* = 4) or development of clonal abnormalities in the Philadelphia-negative cells (*n* = 1).

In total, 6 patients (4 receiving DAS and 2 NIL) asked to stop therapy while in stable DMR: four of them are currently in treatment free remission after a median time of 25 months and two lost MMR and restarted therapy after 4 and 6 months, respectively.

We recorded 3 progressions to ABP in the DAS group and 1 in the NIL group. Sixteen patients in the DAS cohort died for CV events (*n* = 5), progression (*n* = 2), second neoplasms (*n* = 3), infection (*n* = 1) or physical deterioration/other causes (*n* = 5), compared to 9 deaths in the NIL cohort for CV event (*n* = 1), second neoplasms (*n* = 4), or physical deterioration/other causes (*n* = 4). Consequently, 5-year PFS was 85.5% (95% CI: 75.7–91.5) for DAS and 87.6% (95% CI: 75.5–94.0) for NIL (*p* = 0.96) (Figure 3B) and 5-year OS was 87.9% (95% CI: 78.6–93.3) for DAS and 89.1% (95% CI: 77.1–95.0) for NIL (*p* = 0.88) (Figure 3C).

## DISCUSSION

In this study, we analyzed the outcomes of second-line 2G-TKI therapy in a “real life” setting of CML patients failing imatinib. We found higher rates of cytogenetic and molecular responses compared to those reported in the previous studies [4, 5], with a significant percentage of patients achieved a stable deep molecular response; more, we found that DAS and NIL are substantially equivalent and safe.

In patients failing IM, selection of TKI is generally based on the safety profile and patient's concomitant medical conditions, on the presence of BCR-ABL1 mutations or on compliance to treatment [8, 9]. Second generation TKIs have never been compared head-to-head in a prospective clinical trial, neither in the first- nor in the second-line setting, and a comparison of the results reported in the phase 2 studies that led to drugs’ registration are difficult because of different patient selection and protocol criteria [10]. A propensity score matching analysis in the front-line setting demonstrated that DAS and NIL offer similar response rates and survival outcomes [11]. Besides a few cost-effective analyses on second-line treatments after IM failure [12–14], the only experience focused on a comparison between DAS and NIL on clinical outcomes in IM-resistant or intolerant patients came from an online medical chart review of 597 CML patients treated by 122 haematologists and oncologists in the United States [15]. Although NIL was found to be associated with a longer PFS (*p* = 0.03) and a trend toward a better OS (*p* = 0.067), these results may have been biased by various factors, such as the low mean number of patients per physician (less than 5) and a median follow-up of less than 12 months.

With the limits of all retrospective and non-randomized studies, our analysis was carried on in 10 haematological centers from North-Eastern Italy, with experience in CML management, including all consecutive cases fitting inclusion criteria to minimize selection bias.

Our data suggest a similar efficacy of DAS and NIL after IM failure in CP-CML, with high rates of responses and excellent long-term survival. Interestingly, due to the earlier DAS availability In Italy, approximately two years before NIL, in the years 2007-08 we switched to DAS 24 patients compared to only 2 treated with NIL. So, it is arguable that a group of IM-resistant patients, that are known to respond less to 2G-TKIs than the intolerant ones [16], received the first available 2G-TKI, thus unbalancing the two cohorts; nonetheless, the long-term survival of DAS- and NIL-treated patients was almost identical and close to 90%.

Compared to the published data from clinical trials [4, 5], in our real-life experience the rates of CCyR and MMR were higher, around 70% vs 50% and 65% vs 40%, respectively. This may be due to both a longer experience with 2G-TKIs and lack of protocol constraints, enabling a steadier use of the drugs, as confirmed by a significant proportion of patients remaining in DAS and NIL treatment over time. Notably, after non-severe or recurrent toxicities, the 2G-TKI dose was permanently reduced in about one third of our patients, and 90% of them maintained or improved their molecular response after dose reduction, demonstrating the advantage of dose adaptation in clinical practice over the adherence to strict protocol rules. As a consequence, after a median observation time of four years, more than 50% of our patients were still in treatment with their second-line TKI, a figure almost double than those reported by Shah *et al*. for DAS [4] and Giles *et al*. for NIL [5]. Very few patients experienced the potentially life-threatening AEs described for both DAS and NIL [17, 18], as we recorded only one case of PAH under DAS and two cases of PAOD with NIL. More generally, safety profile of both drugs was acceptable, with 15–20% discontinuing therapy due to drug-related adverse events, figures in line with those previously reported for DAS [19] and NIL [5].

Long-term use of 2G-TKI in second-line treatment after IM failure resulted in about 40% of patients achieving the “safe haven” of DMR, with around 60% of them in stable MR4, a strong prerequisite for discontinuing treatment [20]. A recent work focused on the use of DAS or NIL as third-line treatment after failure of 2 previous TKIs showed that 16 out of 21 patients in this setting were able to gain and/or maintain an optimal molecular response and 4 of them stopped the treatment [21]. Though the number of patients who actually attempted discontinuation in our cohort is very small, these data suggest that the goal of treatment-free remission could be pursued also in patients receiving 2G-TKI after IM failure.

To date, there are no clear indications to guide treatment of CP-CML patients failing IM [22]. The most widely used tools are BCR-ABL1 mutational status and patient's comorbidities. At present, however, only a small and definitive number of mutations have been shown to confer insensitivity to DAS (V299L and F317L/V/I/C), NIL (Y253H, E255K/V, and F359I/V/C) or both (T351I)[23], while the percentage of patients for whom a specific concomitant disease may preclude the use of one of the two 2G-TKIs does not exceed 20% [24]. Our study indicates that, in a “real life” setting, both DAS and NIL are equally effective, with high rates of cytogenetic and molecular responses, good persistence on therapy with acceptable toxicity and a significant chance to achieve a stable DMR.

## MATERIALS AND METHODS

We retrospectively analysed a database of consecutive CML patients treated at 10 Italian haematologic centres between January 2007 and December 2015. The inclusion criteria were as follows: (1) age ≥18 years; (2) diagnosis of CP CML; (3) use of DAS or NIL as second-line therapy after resistance or intolerance to IM; (4) no evolution to accelerated or blast phase (ABP) at the time of DAS or NIL start; (5) no detection of BCR-ABL1 mutations known to confer resistance to DAS (V299L and F317L/V/I/C), NIL (Y253H, E255K/V and F359V/I/C) or both (T315I). All patients who met the required criteria were included in the analysis. We compared the characteristics of the two groups at the time of CML diagnosis and at the time of IM failure, including the cause of switch to 2G-TKI, duration of IM therapy, IM dose escalation and Hammersmith score to predict the probability of response to 2G-TKIs [25]. Starting dose of 2G-TKI, causes of prolonged (i.e. lasting ≥1 month) or permanent interruption, and causes of permanent dose reduction were recorded for each patient, along with the dynamics of molecular response upon dose modifications. Cytogenetic and molecular responses were evaluated according to the 2013 ELN recommendations [26]. Major molecular response (MMR) was defined as BCR-ABL^IS^ ratio <0.1%. Deep molecular response (DMR^4^) was defined as BCR-ABL^IS^ ratio ≤0.01% or undetectable disease with ≥10,000 ABL copies, i.e. MR^4^. Patients in MR^4^ lasting ≥2 years with at least a Q-RT-PCR test every 6 months and ongoing at the last follow-up were defined as in stable DMR.

Time to treatment failure (TTF) was calculated from the start of 2G-TKI to the earliest date of any of the following events: progression to ABP, death for any cause at any time, treatment discontinuation for primary or secondary resistance or intolerance. Progression free survival (PFS) was defined as the time from the start of 2G-TKI to ABP or death. Overall survival (OS) was defined as the time from the start of 2G-TKI to the date of death from any cause.

### Statistical analysis

The baseline characteristics of patients and rates of AEs were compared between the groups of NIL- and DAS-treated patients using the Pearson chi-square or Fisher exact test for categorical variables and Mann–Whitney *U* test for continuous variables. TTF, PFS, and OS were calculated using the Kaplan–Meier method, and the values were compared using the long-rank test. All tests were two-sided, and a *p* value < 0.05 was considered statistically significant. All statistical analyses were performed using the EZR package [27].
